# High-Accuracy Prediction of Chunmee Tea Grade via DeepSpectra Model and Near-Infrared Spectroscopy

**DOI:** 10.3390/foods15111848

**Published:** 2026-05-23

**Authors:** Yatong Zhang, Mobing Ren, Xiaohong Wu, Bin Wu

**Affiliations:** 1 Mengxi Honors College, Jiangsu University, Zhenjiang 212013, China; 3230502090@stmail.ujs.edu.cn; 2 School of Computer Science and Communication Engineering, Jiangsu University, Zhenjiang 212013, China; 3230601023@stmail.ujs.edu.cn; 3 School of Electrical and Information Engineering, Jiangsu University, Zhenjiang 212013, China; 4 High-Tech Key Laboratory of Agricultural Equipment and Intelligence of Jiangsu Province, Jiangsu University, Zhenjiang 212013, China; 5 Department of Information Engineering, Chuzhou Polytechnic, Chuzhou 239000, China; 6 School of Computer Science and Engineering, Southeast University, Nanjing 211189, China

**Keywords:** Chunmee tea, near-infrared spectroscopy, multi-scale feature extraction, light gradient boosting machine, convolutional neural network, inception module, end-to-end learning

## Abstract

Chunmee tea quality is critical to its grading, and accurate identification is essential for quality evaluation and market valuation. However, traditional machine learning relies on manual feature extraction and causes spectral information loss, while conventional one-dimensional convolutional neural networks (1D-CNNs) are restricted by fixed kernels and narrow receptive fields, making multi-scale feature capture difficult. In this study, an improved DeepSpectra model integrated with the Inception module and residual connections was proposed for end-to-end automatic grading of Chunmee tea. A total of 360 samples across six grades (60 samples per grade) were collected using an Antaris II near-infrared spectrometer and preprocessed by multiplicative scatter correction (MSC). The proposed model was compared with other models. Results showed that under a 7:1:2 train–validation–test split, the proposed DeepSpectra achieved an average test accuracy of 96.39 ± 1.63% across ten random sample divisions, significantly outperforming the other models (*p* < 0.05). The model also exhibited excellent stability in five-fold cross-validation and superior generalization in small-sample scenarios, and a lightweight structure with low inference latency of 2.2 ms, which is suitable for real-time industrial applications. This work provides a reliable, efficient, and end-to-end method for grading Chunmee tea and offers a promising strategy for intelligent and rapid quality control of green tea.

## 1. Introduction

Tea is the second most widely consumed beverage globally, surpassed only by water [1]. Among its diverse varieties, green tea is particularly esteemed for its distinctive aroma, delicate flavor, and abundance of bioactive constituents, most notably catechins, a predominant class of polyphenols recognized for their antimutagenic, antibacterial, and antiviral properties [2]. These phytochemicals contribute to cancer-preventive and tumor-suppressive activities, highlighting green tea as a functional food [3]. The quality and market valuation of green tea are primarily determined by factors such as cultivar [4], geographical origin [5], and processing techniques [6], which collectively influence its chemical profile and sensory characteristics. Owing to such intrinsic variations, it is of practical significance to develop reliable and objective tea grade classification methods.

Traditionally, tea grading has relied on human sensory evaluation and chemical detection. Human sensory evaluation is an ancient method, dependent on the evaluator’s perception of appearance, aroma, and taste, but training experienced evaluators is costly and time-consuming [7,8]. Chemical detection techniques, such as high-performance liquid chromatography (HPLC) and gas chromatography–mass spectrometry (GC-MS), provide precise quantification of metabolites but are limited in on-site or large-scale applications due to their destructive sampling, laborious procedures, and high equipment costs [9,10,11,12,13]. Although alternative strategies, including Raman spectroscopy, electronic tongue systems, and metabolomics, have shown great potential [14,15,16,17], they still face a dual challenge in balancing detection accuracy with operational efficiency in practical implementation.

In contrast, near-infrared spectroscopy (NIRS) offers a rapid, non-destructive, and high-throughput alternative for quality assessment [18,19,20]. The success of deep learning (e.g., convolutional neural network–long short-term memory (CNN-LSTM)) in agricultural products further validates the feasibility of such technologies [21]. When integrated with machine learning (e.g., support vector machine (SVM) and random forest), NIRS has demonstrated robust performance in tea variety discrimination and grading [22,23,24]. Moreover, feature extraction techniques like partial least squares—discriminant analysis (PLS-DA) remain pivotal in optimizing spectral learning frameworks and enhancing classification accuracy [25,26].

Despite these advancements, conventional machine learning still relies heavily on handcrafted features, which may lead to the loss of informative spectral content. The high dimensionality and redundant nature of NIR spectra often result in the “small sample size problem”, increasing overfitting risks [27,28]. While CNNs offer automated feature extraction [29,30,31], their fixed-scale kernels limit the capacity to simultaneously capture broad trends and fine-scale fluctuations. To address this, the adaptive double weighted gradient boosting decision tree (ADGWDTO) algorithm provides a robust framework for optimizing feature selection and hyperparameters [32], offering a promising approach to mitigate information loss in Chunmee tea grading.

To overcome these limitations, the Inception architecture has been introduced into spectroscopic analysis [33]. By integrating parallel pathways with multiple kernel sizes, this framework enables multi-scale information fusion, significantly enhancing the representation of complex spectral patterns [34]. Inception-based models, such as 1D-Inception-ResNet [35,36] and DeepSpectra [37], have demonstrated superior performance over conventional CNNs in assessing the quality of various agricultural products. Despite this potential, their application to Chunmee tea grading remains largely unexplored, leaving critical research gaps to be addressed.

The main novelties of this study are threefold. First, an improved DeepSpectra network integrating Inception modules and residual connections is proposed. This architecture enables multi-scale spectral feature extraction tailored to capture the subtle discriminant signals of Chunmee tea. Second, the model’s robustness was systematically evaluated under limited sample sizes; it maintained >90% accuracy even with only 30% of the training data. Third, the framework achieves a balance between high precision and industrial efficiency, with an inference latency of 2.20 ms and a compact size of 218.9 k parameters. Together, these contributions establish the first end-to-end, industrially viable NIRS framework for Chunmee tea grade identification.

## 2. Materials and Methods

### 2.1. Sample Preparation

Chunmee tea was selected as the experimental subject for this study. Under GB/T 14456.5–2016 [38], Chunmee tea is categorized into seven grades: Special Grade (Special 1, 2) and Grades 1–5. This study analyzed the six primary grades: SG1, SG2, and G1–G4, excluding G5 due to its lower commercial quality and limited prevalence in target export markets. All tea samples were collected from Hangzhou, China, harvested in spring 2021, and processed strictly in accordance with the national standard GB/T 32742-2016 (Technical specification for Mee tea producing and manufacturing) [39]. All 360 samples were biologically independent and derived from separate raw tea batches without repeated preparation. A total of 360 samples were collected, with 60 per grade [40]. First, approximately 3.0 g of Chunmee tea leaves were weighed using an electronic balance and transferred into a beaker. Subsequently, 150 mL of hot deionized water at 100 °C was poured into the beaker for tea infusion preparation. The mixture was kept static for 4 min without stirring during the brewing process. After cooling naturally to room temperature, the tea residue was removed by filtration with filter paper and a funnel, and the filtrate of tea infusion was retained. Finally, a small amount of the clarified tea infusion was pipetted into a quartz cuvette for subsequent spectral measurement. All samples were graded prior to spectral acquisition following national standard sensory indices: appearance, aroma, taste, and liquor color, ensuring objective and accurate grade labeling.

### 2.2. Data Acquisition

In accordance with China National Standard GB/T 21186-2007 (General Rules for Near-Infrared Spectroscopic Analysis) [41], the laboratory temperature was maintained at 25 ± 1 °C and relative humidity at 50 ± 5% throughout the experiment to guarantee environmental stability. Before spectral acquisition, the Antaris II NIR spectrometer (Thermo Fisher Scientific Co., Waltham, MA, USA) was powered on and allowed to stabilize for one hour. The NIR spectra of Chunmee tea samples were collected in diffuse reflectance mode using an integrating sphere. Each sample was scanned 32 times, and the average diffuse reflectance spectrum was calculated to minimize random noise. Spectral data were collected over the wavenumber range of 10,000–4000 cm^−1^. The Antaris II spectrometer provides a nominal spectral resolution of 4 cm^−1^, while the actual spectral data sampling interval is 3.857 cm^−1^. This interval yields 1557 spectral data points for each sample. Scanning was performed at a sampling interval of 4 cm^−1^ to ensure high spectral resolution. Each tea sample was measured in triplicate, and the mean spectrum was used to construct the model.

### 2.3. Data Preprocessing

The primary interferences in the NIR of Chunmee tea infusions are multiplicative interferences caused by particle scattering and liquid surface reflection. This study conducted comparative experiments using four preprocessing strategies: multiplicative scatter correction (MSC), standard normal variate (SNV), 5-point second-order Savitzky–Golay (SG) smoothing, and second-derivative spectroscopy. To ensure statistical rigor and avoid overestimation from a single random split, all preprocessing comparisons were performed using 10 repeated stratified random splits. The mean test accuracies achieved by DeepSpectra were 96.67 ± 1.99% (MSC, 95% CI [95.25%, 98.09%]), 96.81 ± 2.08% (SNV, 95% CI [95.32%, 98.29%]), 95.83 ± 2.45% (SG), and 97.92 ± 2.10% (SG2), respectively.

As noted by Dhanoa et al. [42], MSC and SNV are mathematically closely related and yield highly correlated outputs when the mean spectrum is used as the reference. In line with this conclusion, MSC and SNV show no statistically significant difference in the present study. Although MSC and SNV perform similarly, MSC is adopted in this study for its stable performance in correcting multiplicative scattering in tea infusion spectra. The slightly higher accuracy (98.15%) reported in Wu et al. [40] using SNV is attributed to a different train–validation–test splitting strategy rather than an inherent advantage of SNV.

To ensure the objectivity and reliability of the model evaluation, the dataset was divided into training, validation, and test sets through stratified random sampling. All preprocessing operations strictly adhered to the principle of being fitted exclusively on the training set, with no information leakage to the validation or test set.

### 2.4. Grade Identification System

Based on the original DeepSpectra framework developed for quantitative regression, this paper proposes a modified DeepSpectra architecture integrating Inception modules and residual connections, which is innovatively employed for multi-class tea grade classification. The workflow of the developed Chunmee tea grade identification framework comprises three key stages: initially, the spectral data of Chunmee tea samples were collected with an Antaris II NIR spectrometer; subsequently, the raw spectra were pretreated using MSC to reduce baseline drift and correct multiplicative scattering effects; and finally, the preprocessed spectral data were subjected to automated feature extraction and classification. A comprehensive set of baseline models is included for a fair comparison: light gradient boosting machine (LightGBM) [43,44], 1D-CNN [45], PLS-DA, SVM (RBF), k-nearest neighbors (KNN), Hybrid CNN-Transformer (Hybrid-TNet) [46], and fuzzy multinomial linear discriminant analysis (FMLDA) + KNN [40]. Based on 10 repeated stratified random splits with mean accuracy ± standard deviation and 95% confidence intervals, DeepSpectra achieved the highest performance (96.39 ± 1.63%), significantly outperforming all baseline models. The results confirm its stronger ability to extract discriminative spectral features and improve classification accuracy. Therefore, integrating DeepSpectra with NIR spectroscopy offers a robust and reliable solution for Chunmee tea grade classification.

### 2.5. LightGBM Model Analysis

In this study, the LightGBM [47] framework was initially employed as a baseline model to assess the separability of Chunmee tea NIR spectra. As a highly efficient gradient boosting architecture based on decision tree learners, LightGBM iteratively minimizes residuals to enhance classification performance. Its superior computational efficiency and inherent suitability for high-dimensional data make it a robust choice for initial spectroscopic analysis.

To handle the non-linear decision boundaries within the MSC-preprocessed and standardized spectra, a multiclass LightGBM model was implemented using a leaf-wise tree growth strategy. The key hyperparameters were meticulously determined based on preliminary empirical trials and practical heuristics to balance model complexity with generalization capacity. Specifically, the learning rate was fixed at 0.05, and the number of leaves was constrained to 31. To mitigate overfitting, a stochastic training strategy was adopted by setting both feature and bagging fractions to 0.8 with a bagging frequency of 5. Furthermore, L1 and L2 regularization terms were both integrated at a coefficient of 0.1 to suppress noise sensitivity.

The model was evaluated over 10 independent stratified random trials, utilizing a 70%/10%/20% split for training, validation, and testing, respectively. To ensure optimal convergence and prevent overfitting, the validation set was utilized to implement an early stopping mechanism, ensuring a desirable balance between bias and variance without the computational overhead of cross-validation during the optimization phase.

However, NIR spectra typically contain fine-grained absorption features, overlapped spectral bands, and considerable noise, which limits the ability of traditional machine learning methods to extract multi-scale and hierarchically discriminative representations automatically. Therefore, to further enhance classification performance, this study introduces deep learning models, particularly CNNs capable of automated hierarchical feature extraction.

### 2.6. 1D-CNN Model Analysis

Compared to conventional machine learning algorithms, CNNs exhibit superior generalization and lower susceptibility to overfitting, thereby improving model robustness and predictive reliability [48]. In recent years, deep learning-based CNN architectures have been effectively employed in tea quality assessment, where their capacity for automatic spectral feature extraction enables more accurate and efficient classification [49].

In this study, a 1D-CNN was developed to classify Chunmee tea grades based on their NIR spectral characteristics. The developed sequential architecture comprises three convolutional blocks and a three-layer fully connected classifier. Each convolutional block included 1D convolution, batch normalization (BatchNorm), ReLU activation, and dropout. To enhance convergence stability and model generalization, BatchNorm was applied after each convolutional layer, and dropout was introduced to mitigate overfitting and improve overall performance.

**Figure 1:**
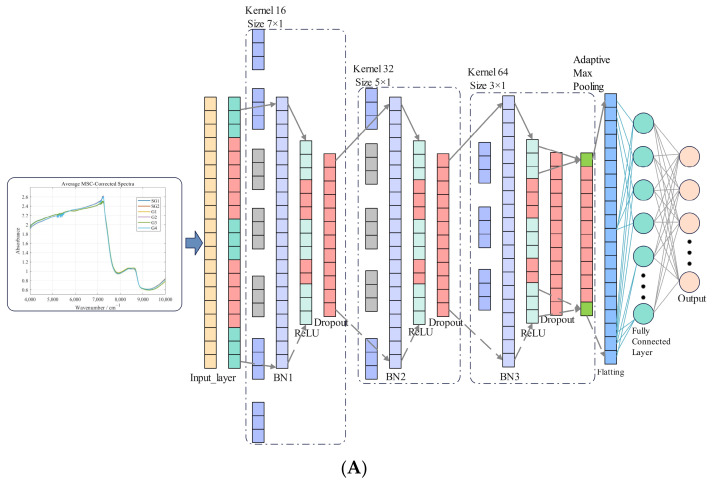

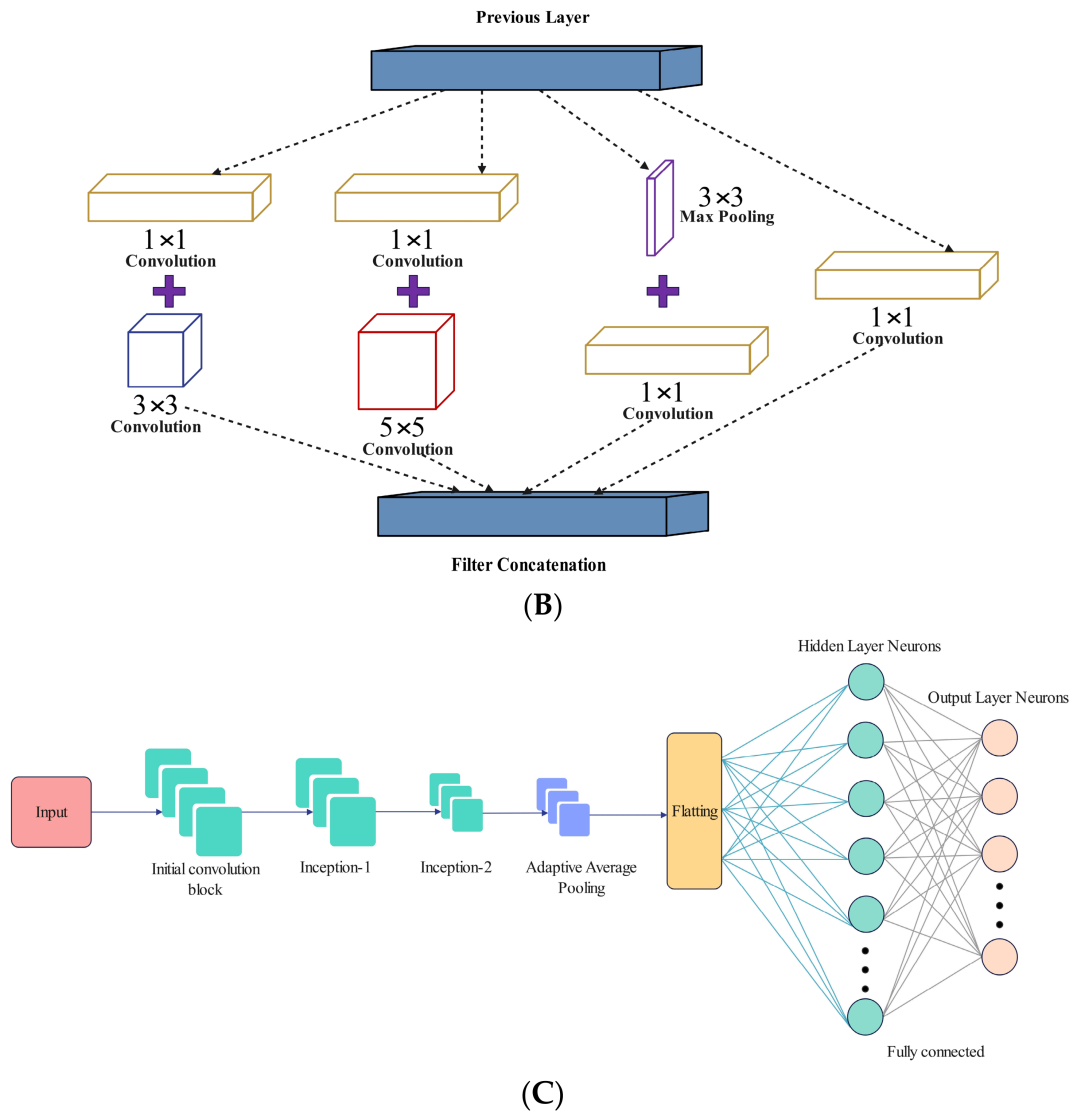
Structural diagrams of different models and modules. (**A**) Structure of the developed 1D-CNN network. Lines indicate multi-path connections; different colors represent distinct operations (yellow = convolution, blue = BN, red = ReLU, green = dropout). Ellipses in the fully connected layer denote variable neuron counts. (**B**) Structure of the Inception module. Dotted lines represent data flow between layers; colored blocks indicate different kernel sizes and corresponding operations. (**C**) Structure of the adopted DeepSpectra network. Solid arrows denote sequential data flow; ellipses indicate omitted hidden neurons in the fully connected layer.

The feature extraction process in the 1D-CNN model begins with a sequence of convolutional blocks designed to capture hierarchical spectral representations. These layers transform the input spectra into a feature data matrix, which is subsequently mapped into fixed-length vector features for each sample. The resulting feature vectors are then passed to the fully connected classifier, which performs the final Chunmee tea grade prediction based on the extracted spectral information. The overall architecture of the 1D-CNN model is illustrated in Figure 1A.

The 1D-CNN model accepts single-channel input signals of variable length, enabling flexible adaptation to diverse spectral datasets. Feature extraction is performed through three successive convolutional blocks with the following detailed parameters: the first convolution had an input channel of 1, output channel of 16, kernel size of 7, and padding of 3; the second convolution had an input channel of 16, output channel of 32, kernel size of 5, and padding of 2; the third convolution had an input channel of 32, output channel of 64, kernel size of 3, and padding of 1. Each convolutional block operated with a stride of 1 to capture both broad and fine-scale spectral features. The resulting feature maps are processed by an adaptive max pooling layer with an output size of 128, which standardizes the output to a fixed spatial dimension. These pooled features are then flattened into an 8192-dimensional vector (derived from 64 channels × 128 time steps). Finally, the three-layer fully connected classifier maps the extracted features through the sequence of 64 × 128 → 512 → 128 → 6, outputting a 6-dimensional logit vector corresponding to the six predefined Chunmee tea grades. To prevent overfitting, dropout rates were set to 0.1 in convolutional blocks and 0.2 in fully connected layers. This architecture was specifically optimized for Chunmee tea grade classification, and its effectiveness was evaluated based on classification accuracy across all Chunmee tea grade categories.

### 2.7. DeepSpectra Model Analysis

Conventional CNNs generally utilize a single-path feature propagation mechanism, which constrains their ability to capture multi-scale spectral information and often leads to overfitting, particularly in applications involving small samples. To overcome these limitations, the Inception module introduces parallel convolutional pathways with diverse kernel sizes, enabling the simultaneous extraction of spectral features at multiple scales. This multi-branch architecture significantly enhances feature representation capacity and model generalization. Building upon this principle, this study employs DeepSpectra, a deep convolutional network architecture specifically optimized for NIR spectral classification. Notably, the original DeepSpectra was designed for quantitative regression with MSE loss. This study adapts the model to six-class tea grade classification by replacing the original MSE loss with cross-entropy loss, incorporating a softmax classifier at the output end, and introducing residual connections along with adjustments to the kernel size and network depth of the Inception branches to boost discriminative performance.

The DeepSpectra architecture is composed of five principal components: an initial convolutional block responsible for low-level feature extraction, followed by two cascaded Inception modules that facilitate hierarchical multi-scale representation learning. These are succeeded by a global adaptive average pooling layer, a flattening layer, and a fully connected classifier that performs the final Chunmee tea grade prediction. To enhance network stability and optimize gradient flow, residual shortcut connections are incorporated throughout the architecture, ensuring the preservation of shallow-layer features and mitigating the problem of gradient vanishing during backpropagation. The four-branch parallel configuration of the Inception module, which enables efficient multi-scale feature fusion, is illustrated in Figure 1B.

The DeepSpectra architecture adopts an end-to-end learning paradigm, allowing direct mapping from raw spectral inputs to final class predictions. Initially, the convolutional block processes the one-dimensional spectral data with an input channel of 1, an output channel of 8, a kernel size of 5, a stride of 2, and a padding of 2. This configuration effectively reduces the sequence length by approximately half while expanding the channel dimension to eight, thereby enhancing the richness of extracted features. The resulting feature maps are then propagated through two sequential Inception modules with residual connections. The first Inception module accepts 8 input channels and outputs 32 channels, using three parallel branches with 1 × 1 convolution, 3 × 3 convolution, 5 × 5 convolution, and max pooling, followed by a 1 × 1 linear convolution layer. The second Inception module takes 32 input channels and outputs 64 channels with the same parallel structure. Subsequently, a global adaptive average pooling layer compresses each feature map into a fixed-length representation of 64, ensuring scale invariance and reducing sensitivity to input variability. The pooled outputs are flattened into a 4096-dimensional feature vector (64 channels × 64 output size), which is then fed into a three-layer fully connected classifier with dimensions of 4096 → 512 → 128 → 6 and dropout rates of 0.2, responsible for generating the final Chunmee tea grade predictions. Collectively, this architecture enables automated feature extraction and high-precision classification directly from NIR spectra, demonstrating the robustness and efficiency of DeepSpectra in tea quality assessment. The complete network structure is presented in Figure 1C.

### 2.8. Model Training and Validation

To ensure training stability and generalization capability, all three models adopted a systematic training configuration. A fixed random seed of 42 was used throughout all experiments to ensure full reproducibility.

To mitigate overfitting, the models integrated multiple regularization techniques. Dropout layers were embedded in the feature extraction module, randomly deactivating 20% of neurons to prevent feature co-adaptation. L2 regularization was introduced in the optimizer to penalize excessive weights. The loss function employed cross-entropy loss with label smoothing (coefficient of 0.1) to alleviate overconfidence in model predictions. An Adam optimizer (PyCharm2024.3.2) was employed with a weight decay coefficient of 1 × 10^−4^, combined with a Warmup-Cosine learning rate scheduling strategy. The learning rate increased linearly during the first 5 epochs (warmup), followed by a cosine decay, balancing convergence in early training and optimization precision in later stages. The training was set for 50 epochs, with an early stopping mechanism (patience of 30) that terminated training when validation accuracy showed no improvement for 30 consecutive epochs, and the optimal model weights were saved.

To comprehensively evaluate the classification performance of the models, this study adopted Accuracy, Weighted Precision, Weighted Recall, and Weighted F1-score as the core evaluation metrics. To ensure statistical robustness, all metrics are reported as mean ± standard deviation across 10 repeated stratified random splits, with 95% confidence intervals (CI) provided to quantify result reliability. Paired *t*-tests were used to verify the statistical significance of performance differences between models, with *p* < 0.05 indicating a significant difference and *p* < 0.01 indicating an extremely significant difference. Specifically, accuracy refers to the proportion of correctly classified samples to the total number of samples. Weighted precision is calculated as the weighted sum of precision for each class, weighted by the number of samples in that class. Weighted recall is the weighted sum of recall for each class, similarly weighted by the sample size per class. The weighted F1-score, as the weighted harmonic mean of precision and recall, provides a comprehensive measure of model performance across all classes.

To ensure consistency and rigor in data partitioning, this study adopted stratified random sampling as the core strategy, employing a two-tiered system of “basic validation + robustness validation”. In the basic validation stage, 360 samples were divided into a training set (252 samples), a validation set (36 samples), and a test set (72 samples) according to a 7:1:2 ratio using stratified random sampling. The stratification was based on the proportion of samples across six grades, ensuring consistent grade distribution within each dataset. In the robustness validation stage, 10 repeated random splits and 5-fold stratified cross-validation were further applied to eliminate random errors from a single data split. Specifically, the 360 samples were evenly divided into 5 folds (each containing samples from all six grades), with one fold used as the test set in turn and the remaining four folds combined as the training/validation set for experimentation. The necessity of stratified sampling lies in preventing potential grade distribution imbalances caused by random sampling, effectively avoiding model overfitting to grades with high sample sizes and ensuring the reliability of experimental results.

## 3. Results

### 3.1. Spectral Data Analysis and Preprocessing

**Figure 2:**
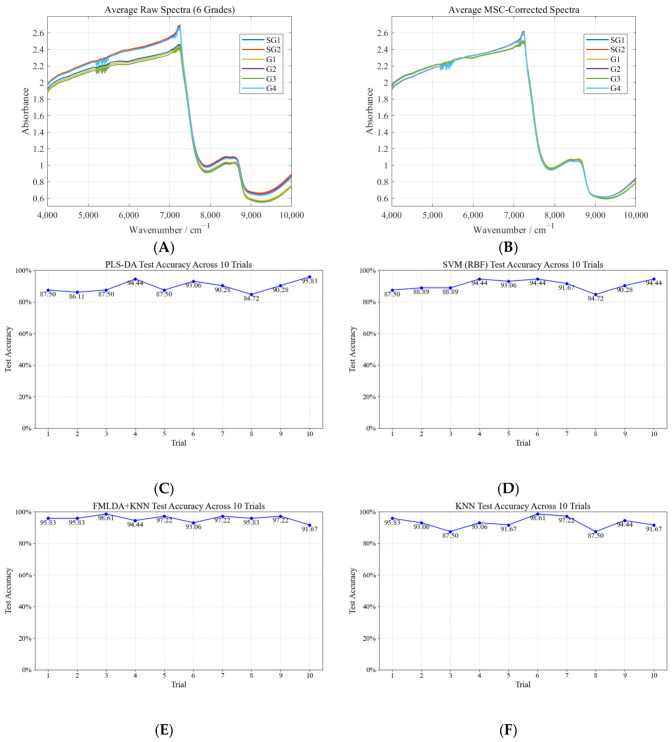

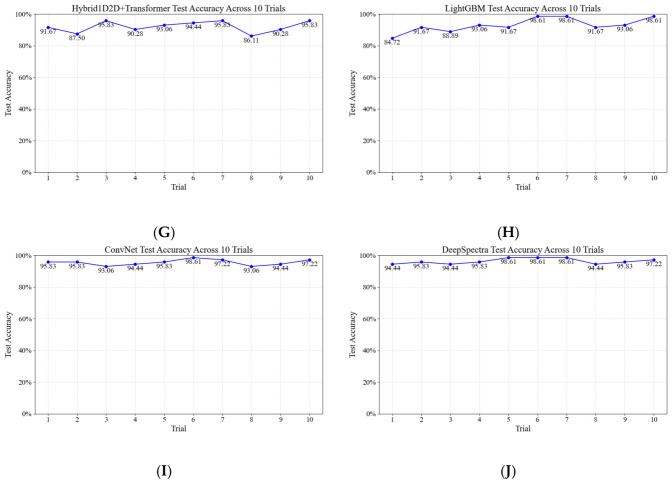
Images of spectral data preprocessing and training processes of all models. I have revised it. (**A**,**B**) denote the average raw NIR spectra and MSC-preprocessed spectra of tea samples at each grade. (**C**–**J**) illustrate the training processes of PLS_DA, SVM_RBF, KNN, FMLDA_KNN, Hybrid-TNet, LightGBM, 1D-CNN and DeepSpectra under ten random data splits. The blue line represents the test accuracy of the model under ten stratified random data splits.

As illustrated in Figure 2A, the average NIR spectra of the six grades exhibit a high degree of similarity in their wavenumber-absorbance distributions, reflecting their comparable chemical compositions. Prominent absorption peaks are observed primarily within the 7100–7300 cm^−1^ region, which corresponds to the first overtone of O–H and N–H stretching vibrations associated with tea polysaccharides and other hydrogen-containing functional groups. In contrast, the 5200–5500 cm^−1^ region displays several spectral spikes, indicative of noise and scattering interferences likely arising from variations in particle size, surface morphology, and light scattering among samples.

Preprocessing of the NIR spectral data of Chunmee tea samples was conducted to reduce scattering effects and enhance overall spectral quality. In this study, MSC, a widely recognized method for compensating multiplicative and additive scattering distortions in NIR spectra, was employed as the primary spectral pretreatment technique. The average MSC-corrected spectra of the six grades, illustrated in Figure 2B, demonstrate a marked reduction in noise within the 5200–5500 cm^−1^ region and a substantial mitigation of light scattering–induced distortions, thereby yielding smoother and more chemically representative spectral profiles suitable for subsequent modeling.

### 3.2. Classification Using LightGBM

In this study, a LightGBM model was developed and systematically optimized for the classification of NIR spectra of Chunmee tea. The training procedure began by feeding the preprocessed and standardized spectral features into the LightGBM classifier. Afterward, the model iteratively partitioned the feature space to construct decision rules and update tree structures based on the gradient information. Leveraging a leaf-wise growth strategy, LightGBM effectively learned nonlinear decision boundaries by optimizing split thresholds, with multi-class cross-entropy as the objective function. Instead, we performed 10 independent stratified random split experiments, training a separate LightGBM model for each trial, and recording the test accuracy for each. The mean, standard deviation, and 95% confidence interval were computed over these 10 trials.

According to the hyperparameter sensitivity analysis, the optimal configuration consisted of 31 leaves, a learning rate of 0.05, 200 boosting iterations, a feature sampling ratio of 0.8, and L1 and L2 regularization coefficients both set to 0.1. This parameter setting produced a well-balanced trade-off between bias and variance, allowing the model to capture subtle nonlinear patterns within the spectral data. To ensure statistical reliability, evaluation was performed using 10 repeated stratified random splits (7:1:2 train/validation/test, 252/36/72 samples). The optimized LightGBM achieved a mean test accuracy of 93.06 ± 4.54% with a 95% confidence interval [89.81%, 96.330%].

### 3.3. Classification Using 1D-CNN

CNNs provide an effective framework for automatic feature extraction and classification from grid-structured data. The core principle involves learning a set of convolutional kernels that slide across the input domain to capture local patterns and hierarchical representations. In this study, a one-dimensional CNN was implemented to process the spectral data of Chunmee tea. The CNN operated as follows: first, a convolutional kernel *W* ∈ *R^k^* was learned to extract specific local features from the one-dimensional spectral input. The kernel parameters were iteratively optimized during training via backpropagation. The kernel then slid over the input spectrum *X* with a stride of 1, performing the convolution operation *F*(*i*) = (*X* × *W*)(*i*) + *b*. The resulting feature maps were processed through a sequential module consisting of batch normalization, the ReLU activation function, and dropout, which together enabled nonlinear transformation and facilitated high-level feature extraction. An adaptive max pooling layer was subsequently applied to compress the spatial dimension of the feature maps to a fixed length of 128, mitigating the impact of variations in input length. The feature tensor was then transformed into a one-dimensional vector after flattening and delivered to a classifier composed of three fully connected layers, mapping abstract features to class probabilities.

To further optimize the model performance, a comprehensive hyperparameter tuning was conducted, with validation accuracy as the core selection criterion. The optimal hyperparameter configuration consisted of a batch size of 8, a dropout rate of 0.2, a label smoothing factor of 0.1, a learning rate of 0.0005, and a weight decay of 0.0001, at which the model achieved the maximum average validation accuracy. Based on 10 repeated stratified random splits, the 1D-CNN achieved a mean test accuracy of 95.83 ± 1.85% (95% CI [94.51%, 97.16%]), significantly outperforming LightGBM. The slight overfitting tendency observed in the late training stage is due to the fixed convolutional kernel structure of 1D-CNN, which cannot fully capture the complex multi-scale spectral features of Chunmee tea, and the model’s generalization ability needs to be further improved.

### 3.4. Classification Using DeepSpectra

At the CNN feature extraction stage, the Chunmee tea samples preserve substantial information from the original NIR spectral data, yet they also encompass redundant features and spectral noise, which can negatively impact model performance. Direct utilization of such high-dimensional inputs for classification increases the risk of overfitting and may impede model convergence. To enhance the classification accuracy of Chunmee tea grades, this study integrated the Inception module into the network architecture, enabling the extraction of discriminative and hierarchically representative features from the preprocessed spectra. Within the experimental framework, the Inception module captured multi-scale spectral features by employing parallel convolutional kernels of varying receptive fields, specifically, 1 × 1 + 3 × 3, 1 × 1 + 5 × 5, and max pooling operations. This multi-branch design effectively augmented the representational capacity of the network and substantially improved its generalization performance across diverse Chunmee tea grades.

Before convolutional processing, multi-scale spectral features were extracted through an Inception-based architecture. Specifically, the raw spectral data *X* ∈ *R*^*N* × 1557^ were first standardized and reshaped into a three-dimensional tensor *X_in_* ∈ *R*^*N* × 1 × 1557^, which satisfied the input specifications of one-dimensional convolutions. This tensor was then passed through a feature extraction network composed of two cascaded Inception modules. The resulting high-level feature maps *F* ∈ *R*^*N* × 64 × 64^ were compressed into a 4096-dimensional feature vector *Z* ∈ *R*^*N* × 4096^ via adaptive average pooling and flattening, which was subsequently passed to a fully connected classifier for the final classification.

According to the hyperparameter tuning, the final optimal hyperparameter configuration consisted of a batch size of 8, a dropout rate of 0.2, a label smoothing factor of 0.2, a learning rate of 0.001, and a weight decay of 0.0005, under which the model achieved the maximum average validation accuracy. Based on 10 repeated stratified random splits, DeepSpectra achieved a mean test accuracy of 96.39 ± 1.63% with a 95% confidence interval [95.22%, 97.56%], significantly outperforming 1D-CNN, LightGBM, and FMLDA+KNN. The consistent trend of training and test accuracy curves indicates that the regularization strategies and Warmup-Cosine learning rate scheduling effectively mitigate overfitting. Paired *t*-tests showed that the accuracy of DeepSpectra was significantly higher than that of 1D-CNN (*p* < 0.05), confirming the superiority of multi-scale feature extraction based on the Inception module.

### 3.5. Classification Using PLS_DA, SVM_RBF, KNN, FMLDA_KNN, Hybrid-TNet

To rigorously evaluate the competitive advantage of the proposed DeepSpectra model, we conducted a comparative analysis against established baseline methods and a newly integrated deep learning architecture, Hybrid-TNet (a hybrid 1D-2D CNN with Transformer). The baselines include PLS-DA, SVM (RBF kernel), KNN, and the recently proposed FMLDA. All models were evaluated under the same experimental framework, using identical 10-trial random stratified splits and preprocessing protocols.

The experimental results demonstrate that while traditional linear and non-linear classifiers achieve respectable performance, they exhibit specific limitations. PLS-DA (89.72%) and SVM (90.83%) provide a solid baseline but struggle to capture the complex, fully non-linear latent variables within the highly overlapping spectra of Chunmee tea. In addition, the revised average accuracy of FMLDA+KNN across 10 trials of stratified random splitting is 95.69%, which is slightly lower than the 98.15% reported by Wu et al. [40]. This discrepancy originates from inconsistent data partitioning strategies. To ensure a fair experimental comparison with the DeepSpectra model, we adopted 10 trials of standardized stratified random splitting with fixed random seeds. By contrast, Wu et al. [40] employed a non-standard partition scheme that manually split samples by category order without fixed random seeds.

To further explore the potential of deep learning, we also implemented Hybrid-TNet. While Hybrid-TNet achieved high accuracy (91.81% ± 2.46%), it highlighted the different trade-offs in spectral analysis: the Transformer’s self-attention mechanism excels at capturing long-range dependencies, yet requires careful tuning in relatively small tea datasets to avoid overfitting.

In contrast, our proposed DeepSpectra model outperforms these benchmarks. The superiority of DeepSpectra can be attributed to its Inception-based multi-scale feature extraction, which autonomously captures subtle discriminant signals that handcrafted or even more complex attention-based architectures might over-engineer for this specific task. Furthermore, the significantly lower standard deviation of DeepSpectra underscores its superior robustness and generalization capability across various random samplings, confirming its efficacy as an advanced tool for industrial tea quality assessment.

### 3.6. Five-Fold Cross-Validation Results

**Figure 3:**
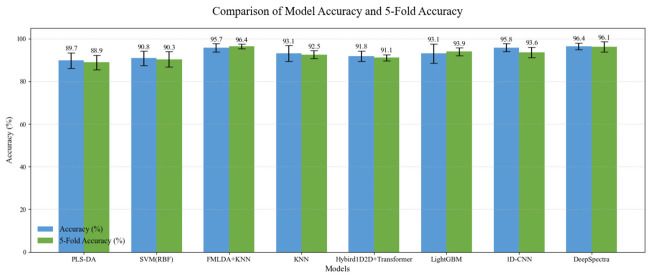
Accuracy of all models under ten random data splits and 5-fold cross-validation.

To eliminate the random errors caused by a single data split and further validate the model’s generalization ability, 5-fold stratified cross-validation was used to evaluate model performance rigorously. As shown in Figure 3, the DeepSpectra model achieved the best performance, with average test accuracy, weighted precision, weighted recall, and weighted F1-score of 96.11% ± 2.39%, 96.10% ± 2.39%, 96.11% ± 2.39%, and 96.10% ± 2.39%, respectively. The term “weighted average” refers to an average where each class’s contribution is weighted by its sample size [50]. The marginal difference in average accuracy between random splitting and cross-validation was extremely small, and both methods yielded low standard deviations. This demonstrates that the model maintains stable and reliable classification performance, with negligible performance fluctuation caused by different sample partitioning strategies, further verifying its good robustness and generalization ability.

Compared with the other models, DeepSpectra showed significant improvements in all metrics and the smallest standard deviation, demonstrating the strongest robustness and generalization ability. The 1D-CNN outperformed LightGBM but exhibited slightly lower stability than DeepSpectra. In contrast, the LightGBM model, which relied on manual feature engineering, failed to effectively capture multi-scale deep spectral features, resulting in the lowest performance and the largest fluctuation.

Although the FMLDA+KNN method achieves a competitive accuracy on the same dataset, its performance is highly sensitive to the hyperparameter m. The optimal result is only obtained at m = 2.7 (where m > 1 in general), and any slight deviation of m will cause an obvious drop in classification accuracy. Notably, this optimal m = 2.7 is purely obtained by trial-and-error enumeration, and there is no mature optimization algorithm or theoretical derivation to automatically solve or predict the optimal hyperparameter. In contrast, the proposed improved DeepSpectra model avoids manual hyperparameter tuning dependence, exhibits superior robustness and generalization ability under sample partitioning and small-sample conditions, and delivers stable and reliable performance without relying on empirical parameter adjustment. Therefore, beyond mere accuracy comparison, the core contribution of this work lies in constructing an end-to-end intelligent grading framework with stronger robustness, higher practicability, and better industrial applicability, rather than relying on empirically determined optimal hyperparameters. The comparison results of ten stratified random splits and 5-fold cross-validation for all models are shown in Figure 3. The prediction accuracy with 95% confidence intervals from ten stratified random splits and the results of 5-fold cross-validation are listed in Table 1.

### 3.7. Small-Sample Performance Results

To verify the applicability of the model in small-sample industrial scenarios (training sample size ≤ 50% of the original dataset), this study evaluated the performance of three models under three training sample ratios: 100% (original data), 50%, and 30%.

When the training sample is reduced to 50%, the accuracy of DeepSpectra remains at 94.72 ± 2.04%, with only a 2.5-percentage-point drop. When the training sample is further reduced to 30%, DeepSpectra still achieves 91.11 ± 4.78%, significantly higher than other models.

These results demonstrate that the Inception-based multi-scale feature-extraction architecture and the residual-connection design of DeepSpectra can fully exploit spectral features from limited samples. This design effectively mitigates overfitting in small-sample scenarios, making the model better suited for practical industrial production with insufficient sample availability.

**Figure 4:**
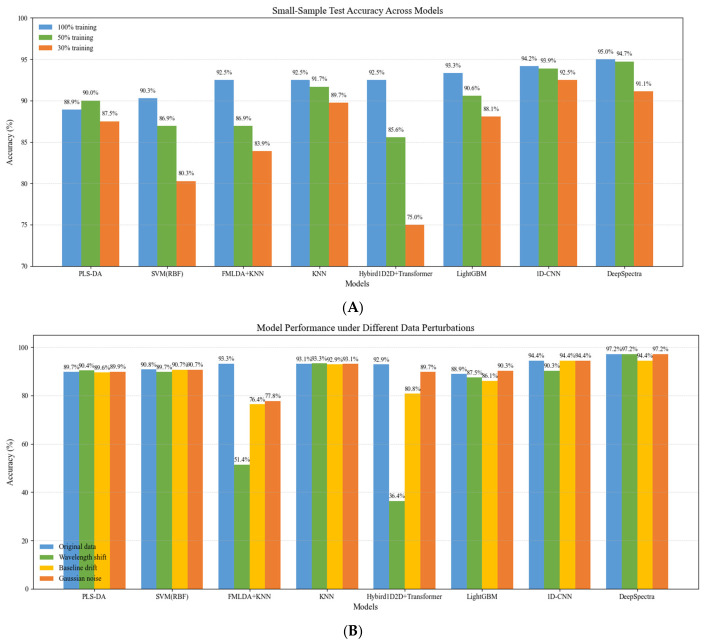
Distribution of small-sample test results and robustness test results for all models, including wavelength shift (5 points), baseline drift (±0.1), and Gaussian noise (SNR = 30 dB).

To further enhance the practical industrial applicability of the proposed model, the robustness of the eight models against real-world industrial variables (e.g., instrument drift and environmental noise) was additionally evaluated based on a single random data split. Specifically, four disturbance scenarios were designed: wavelength shift (5 points), baseline drift (±0.1), Gaussian noise (SNR = 30 dB), and original data (as the control group). The comparison results of small-sample tests and robustness verification for all models are presented in Figure 4. The corresponding test results are summarized in Table 2.

Notably, DeepSpectra exhibited no accuracy degradation under wavelength shift and Gaussian noise disturbances, and only a 2.78-percentage-point drop under baseline drift. This superior robustness is attributed to the multi-scale feature extraction capability of the Inception module, which can effectively suppress interference from industrial variables and retain key discriminative spectral features. Combined with its excellent few-shot learning performance, DeepSpectra fully demonstrates strong adaptability to practical industrial scenarios with limited samples and complex interference.

## 4. Discussion

### 4.1. Model Performance Comparison and Superiority Analysis

Table 1 summarizes the performance metrics of all evaluated models, including traditional machine learning methods and deep learning models, showing the mean test accuracy based on 10 repeated stratified random splits, 5-fold cross-validation accuracy, and F1-score. This performance underscores the effectiveness of integrating multi-scale feature extraction and residual learning within DeepSpectra, which collectively enhanced the model’s generalization ability, feature discrimination capacity, and overall classification performance for NIR spectral data of Chunmee tea. Traditional machine learning methods rely on manual feature extraction and shallow modeling, limiting their ability to capture subtle nonlinear spectral differences. In comparison, the proposed DeepSpectra model achieves the highest accuracy and the best stability, demonstrating its clear advantages for high-precision tea grading.

The outstanding advantage of the multi-scale Inception module lies in its high compatibility with the characteristics of tea NIR spectra. The spectra have broad and overlapping absorption peaks, weak inter-grade differences, and coexisting global trend changes and local fine fluctuations. A single-scale convolution kernel can only capture fixed-size features and cannot fully perceive such multi-scale information. In contrast, the multi-branch parallel convolution in the Inception module can simultaneously capture global spectral shape features and local subtle absorption changes, which enables the model to effectively mine weak but critical discriminant information hidden in the spectrum, thereby significantly improving classification accuracy.

The characteristic absorption bands of Chunmee tea at 7100–7300 cm^−1^ are associated with O–H and N–H stretching vibrations, which reflect the content of tea polyphenols, tea polysaccharides, and amino acids—key components determining tea grade. The 5200–5500 cm^−1^ region is easily disturbed by scattering noise, which was effectively eliminated by MSC pretreatment, thereby enhancing spectral quality and model accuracy. The Inception module benefits most significantly from the spectra with similar grades and weak differences. After noise and scattering interference are removed, the spectral curves become smoother, and the real grade-related differences are retained. On this clean input, the multi-scale mechanism can fully exert its feature extraction ability, especially in the weak-difference region of 5200–5500 cm^−1^ and the characteristic absorption band of 7100–7300 cm^−1^, greatly improving the separability of subtle features.

Tea near-infrared spectra contain both narrow characteristic absorption peaks and global waveform variations. Single-scale convolution kernels can only extract features at a fixed receptive field, failing to simultaneously capture local subtle absorption peaks and overall spectral trends. In contrast, multi-scale convolution kernels with different receptive fields can simultaneously mine local fine spectral features and global waveform patterns, adaptively focusing on discriminative bands related to tea biochemical components such as tea polyphenols and amino acids. This multi-granularity feature extraction effectively suppresses noise interference and enhances feature representation, thereby significantly improving the classification performance of tea spectra.

DeepSpectra has the fewest adjacent-grade misclassifications, as its Inception module captures fine-scale absorption peak intensity changes related to grade differences, while residual connections preserve shallow spectral features, making the model more sensitive to subtle spectral discrepancies. In comparison, conventional machine learning models rely on manual feature engineering or shallow statistical modeling, occasionally leading to non-adjacent grade misclassifications (e.g., G2 and G4).

Notably, to address the overfitting risk of DeepSpectra’s high model capacity, this study integrated 10 repeated stratified random splits, 5-fold cross-validation, and multiple regularization strategies. This combined approach effectively mitigated overfitting, ensuring good generalization to unseen data and reducing sensitivity to random training set fluctuations—key to DeepSpectra’s stable and high-precision classification performance.

To further evaluate the influence of the training–testing split ratio on feature extraction and model generalization, experiments were performed using two partition schemes: 8:1:1 (288/36/36) and 7:1.5:1.5 (252/54/54). The corresponding results are summarized in Table 3. All models maintain stable performance under different splits, and DeepSpectra consistently achieves the highest accuracy in all settings. It showed the largest accuracy improvement under the 7:1.5:1.5 ratio, as its strong multi-scale feature learning allowed full use of validation set information to optimize parameters. This consistent superiority across split ratios indicates DeepSpectra’s high stability and adaptability, unaffected by data partitioning methods.

Furthermore, robustness tests under Gaussian noise, baseline drift, and wavelength shift show that DeepSpectra maintains stable accuracy, demonstrating strong resistance to real-world industrial interference. These findings validate that the Inception-based DeepSpectra framework significantly enhances multi-scale spectral feature learning from NIR data. In conclusion, the integration of MSC preprocessing with the DeepSpectra architecture provides a robust and accurate solution for the identification and classification of Chunmee tea grades.

### 4.2. Model Interpretability and Activation Mapping

**Figure 5:**
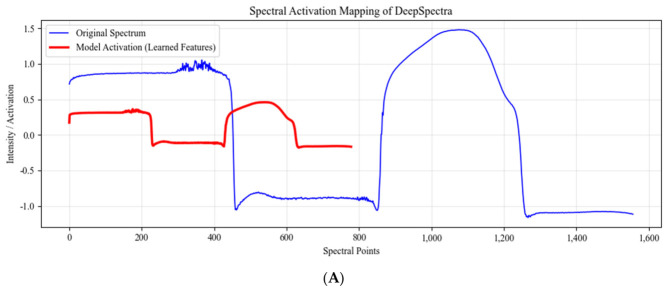

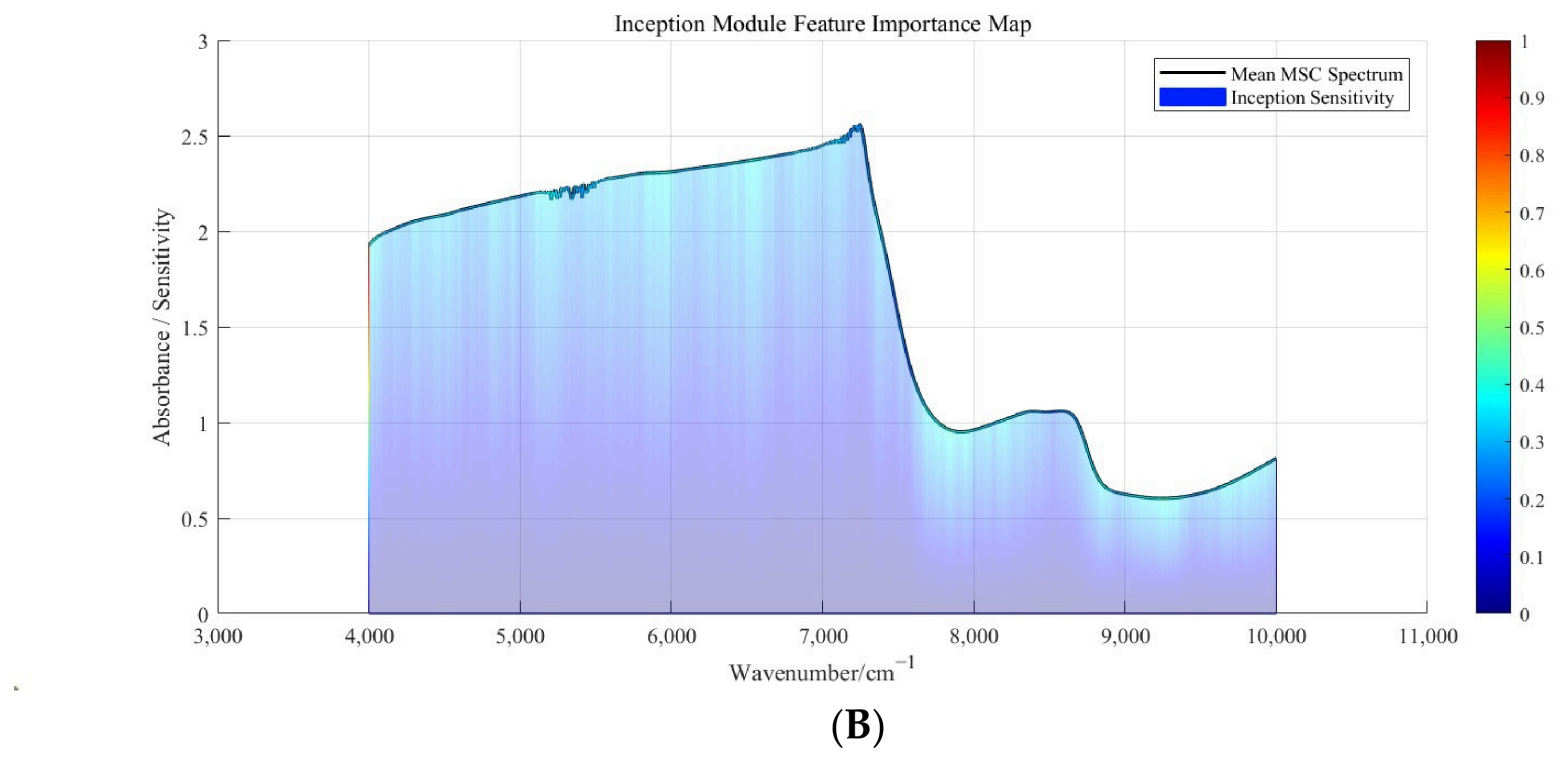
Visualization of learned features by DeepSpectra. (**A**) Spectral activation mapping, where the blue line shows the original spectrum and the red line indicates the model activation, highlighting key regions. (**B**) Inception module feature importance map, with the black line representing the mean MSC spectrum and the color bar denoting sensitivity to different wavenumbers.

To further explore the learned features of the model, a visualization analysis was conducted based on the Inception module activation mapping. The DeepSpectra model automatically extracts local absorption peaks, waveform changes, and subtle discriminative features through multi-scale convolution kernels. The activation map illustrates that the model does not rely on the full spectrum uniformly, but focuses on key wavelength regions highly related to tea polyphenols, amino acids, and carbohydrates, while suppressing interference from redundant bands and noise. Notably, strong activation responses appear at approximately 400 and 600 wavelength points, corresponding to the characteristic absorption regions of chemical components in tea. This confirms that the model captures physically and chemically meaningful features rather than random noise. The interpretability and activation mapping of the DeepSpectra model are shown in Figure 5.

### 4.3. Engineering Application Analysis

To verify the industrial application potential of the models, this section systematically compares the engineering adaptability of LightGBM, 1D-CNN, and DeepSpectra from computational cost, scalability, and deployment feasibility, with key quantitative indicators and hardware configuration parameters summarized in Table 4.

Computational cost is a core constraint in industrial applications, determined by model complexity and inference efficiency. DeepSpectra (218.90 × 10^4^ parameters, 0.02 G FLOPs) has moderate complexity; compared with 1D-CNN (427.04 × 10^4^ parameters, 0.02 G FLOPs), both are lightweight models suitable for industrial scenarios. All models meet real-time detection requirements (<100 ms/sample), among which DeepSpectra achieves a single-sample inference latency of 2.20 ms and a batch-32 latency of 0.07 ms, which can satisfy the high-throughput demands of tea quality grading. DeepSpectra has good scalability; within the batch size range of 1–64, its inference speed increases linearly with the batch size, making it suitable for both centralized laboratory detection and online industrial grading. The model can be deployed on portable spectrometer terminals (single-sample latency < 3 ms) and continuous production lines, and maintains low latency even when running on general-purpose CPUs; LightGBM can serve as a low-power backup solution for edge terminals with limited computing resources.

### 4.4. Feature Extraction Mechanism Comparison

The DeepSpectra model achieves a significant accuracy improvement in Chunmee tea grade classification compared with conventional machine learning methods (PLS-DA, SVM, KNN, FMLDA+KNN) and mainstream deep learning models (LightGBM, Hybrid1D2D+Transformer, 1D-CNN). The performance gap mainly lies in the inherent limitations of existing approaches; most traditional methods rely heavily on manual feature engineering, while standard CNNs adopt single-scale feature extraction, both failing to fully capture complex multi-scale spectral patterns of tea samples. In contrast, DeepSpectra’s end-to-end multi-scale feature learning ability makes it more suitable for the high-dimensional, redundant, and weak difference characteristics of Chunmee tea spectral data.

The higher classification accuracy achieved by DeepSpectra compared with LightGBM and 1D-CNN essentially arises from fundamental differences in feature extraction mechanisms and architectural design. As a traditional machine learning method, LightGBM only captures shallow spectral relationships via manual feature selection and fails to handle high-dimensional redundancy, resulting in the loss of subtle tea-grade-related features. By contrast, DeepSpectra enables end-to-end feature learning without manual engineering and automatically extracts multi-scale deep features adapted to the high-dimensional complexity of Chunmee tea spectra. Unlike 1D-CNN with fixed convolutional kernels and a single feature path that limits feature capture and causes information loss, DeepSpectra uses an Inception module with multi-branch parallel convolutions for full-scale feature extraction and residual connections to preserve shallow features, greatly improving feature representation.

In comparison with existing studies and applications, this work presents three distinct scientific novelties and original contributions to tea quality assessment using NIR spectroscopy. First, this study systematically adapts the original DeepSpectra architecture and systematically validates its feasibility for the NIR spectral recognition of Chunmee tea grades, with a specific focus on overcoming the challenge of extremely weak spectral differences between adjacent grades—an issue that prior applications of DeepSpectra to other agricultural products did not address. Second, targeting the high-dimensional and redundant characteristics of Chunmee tea spectral data, the study innovatively integrates the Inception module with residual connections into the DeepSpectra architecture, which effectively addresses the problem of limited receptive fields in conventional 1D-CNN models and significantly improves the model’s generalization capability under small sample conditions. Third, a standardized and efficient Chunmee tea grading pipeline combining MSC preprocessing and DeepSpectra modeling is constructed in this study. Compared with existing spectral grading methods for tea, this pipeline achieves prominent statistical accuracy gains without eliminating the need for complex feature engineering steps, making it more suitable for practical industrial applications with high efficiency and low operational costs. Furthermore, this standardized grading procedure features low operational complexity and high detection speed (less than 1 min per sample), which well matches the large-scale and high-throughput detection requirements of the industry and presents greater practical application value than existing spectral grading methods.

These original improvements not only advance the technical means of NIR spectral analysis for Chunmee tea grade identification but also provide a novel methodological reference for the spectral quality assessment of other agricultural products. In addition, the above research results verify the feasibility and superiority of the integration of DeepSpectra and NIR spectroscopy in Chunmee tea grade identification, which provides new methods and ideas for the high-precision and automated detection of tea quality.

## 5. Conclusions

This study verifies that the DeepSpectra model integrated with the Inception module can efficiently capture the multi-scale spectral features of Chunmee tea, overcoming the limitations of conventional 1D-CNN and machine learning methods, including PLS-DA, SVM (RBF), KNN, Hybrid-TNet, and FMLDA+KNN in NIR spectral classification for Chunmee tea grade identification. Based on 10 repeated stratified random splits, the adapted DeepSpectra achieves a stable mean classification accuracy of 96.39 ± 1.63% with a 95% confidence interval of [95.22%, 97.56%], consistently outperforming 1D-CNN and LightGBM in overall performance, with statistically significant performance differences (*p* < 0.05).

From an industrial perspective, the grading framework combining DeepSpectra with NIR spectroscopy exhibits strong practicality and feasibility for on-site deployment. It dispenses with complex manual feature engineering and features a standardized modeling workflow, allowing for rapid adaptation to the quality detection demands of tea manufacturing enterprises. Moreover, the model is built on 1D spectral data with low computational complexity, which facilitates its lightweight deployment on portable analytical devices for real-time detection scenarios. Most importantly, its stable and reliable prediction accuracy even under small-sample conditions makes it highly applicable for the rapid screening of tea samples from different production batches and geographical origins in actual industrial production.

In addition, this research confirms the effectiveness of MSC in preprocessing NIR spectral data of Chunmee tea, which can significantly alleviate the negative impacts of light scattering and baseline drift on the performance of subsequent classification models.

Nevertheless, this study still has several limitations that deserve further improvement. First, only Chunmee tea samples from a single production region were included in the experiment, resulting in insufficient sample diversity. The generalization ability of the model for tea samples from other geographical origins needs to be further verified. Second, this study only adopts the MSC pretreatment method; other spectral preprocessing algorithms and their combined strategies were not systematically compared and explored. Third, the model validation was implemented only under laboratory-controlled conditions, and field tests in complex factory environments containing noise, temperature drift, and instrument variation are still lacking. Due to the insufficient sample diversity, the direct industrial generalization and deployment of the proposed model are significantly constrained. Future work will expand the sample coverage of multiple producing areas, screen optimal combined preprocessing schemes, and carry out on-site industrial testing to further improve the generalization, interpretability, and practical deployment capability of the model.

Overall, the integration of DeepSpectra with NIR spectroscopy establishes a robust and efficient framework for Chunmee tea grade classification, providing a promising strategy for the intelligent and rapid quality control of green tea. Unlike previous NIRS-based tea grading studies that rely on manual feature engineering or single-scale CNNs, the key contribution of this study lies in solving the specific problem of subtle spectral differences between adjacent tea grades through an Inception-based multi-scale architecture, by adapting the original DeepSpectra framework originally designed for spectral regression to tea grade classification, as well as quantifying the model’s robustness under extreme sample limitations, a realistic industrial constraint that is often overlooked in existing research. The validated lightweight structure of the proposed model further enables real-time deployment, bridging the gap between laboratory demonstration and practical industrial application.

## 6. Research Prospects

From a scientific research perspective, the sampling scope of Chunmee tea can be further expanded in future studies to cover more geographical origins and production batches. Expanding sample diversity will help enhance the model’s reliability and visual identification ability for Chunmee tea grading. The application scope of the model can be further extended to a wider variety. The DeepSpectra model can be generalized into a universal quality evaluation system applicable to various green tea types.

On this basis, to promote the practical application of this technology, the DeepSpectra model can be further optimized through lightweight network compression and deployed as an embedded algorithm in portable near-infrared spectrometers. This will support rapid on-site grading during tea picking, purchase, processing, and circulation. Furthermore, the application scenarios of the proposed model can be extended to the non-destructive detection of other food products. By leveraging its spectral analysis framework, this model can be applied to the dynamic evaluation of refrigerated kiwifruit quality [51] and the rapid screening of heavy metal contents in agricultural commodities [52].

Ultimately, a universal spectral recognition model can be established, fundamentally promoting the upgrading of the modern tea industry towards digitization, standardization, and intelligence.

## Figures and Tables

**Table 1:** Performance comparison of different models (Mean ± SD, 10 trials).

Model	Accuracy (%)	95% CI	5-Fold Accuracy(%)	F1-Score(%)
PLS-DA	89.72 ± 3.72	[87.06%, 92.38%]	88.89 ± 3.40	88.73 ± 3.43
SVM(RBF)	90.83 ± 3.35	[88.44%, 93.23%]	90.28 ± 3.62	90.23 ± 3.66
FMLDA+KNN	95.69 ± 2.01	[94.26%, 97.13%]	96.39 ± 1.11	96.38 ± 1.11
KNN	93.06 ± 3.70	[90.41%, 95.71%]	92.50 ± 1.88	92.49 ± 1.85
Hybrid-TNet	91.81 ± 2.46	[90.03%, 93.59%]	91.11 ± 1.42	91.03 ± 1.41
LightGBM	93.06 ± 4.54	[89.81%, 96.30%]	93.89 ± 1.88	93.90 ± 1.82
1D-CNN	95.83 ± 1.85	[94.51%, 97.16%]	93.61 ± 2.42	93.51 ± 2.56
DeepSpectra	96.39 ± 1.63	[95.22%, 97.56%]	96.11 ± 2.39	96.10 ± 2.39

**Table 2:** Classification accuracy of different models under industrial interference conditions for robustness evaluation (%).

Model	Original Data	Wavelength Shift (5 Points)	Baseline Drift (±0.1)	Gaussian Noise (SNR = 30 dB)
PLS-DA	89.72	90.42	89.58	89.86
SVM(RBF)	90.83	89.72	90.69	90.69
FMLDA+KNN	93.26	51.39	76.39	77.78
KNN	93.06	93.33	92.92	93.06
Hybrid-TNet	92.92	36.39	80.83	89.72
LightGBM	88.89	87.50	86.11	90.28
1D-CNN	94.44	90.28	94.44	94.44
DeepSpectra	97.22	97.22	94.44	97.22

**Table 3:** Accuracies of the Chunmee tea grade recognition system under different numbers of training/validation/test sets.

Training/Validation/Test Set	Model	Accuracy (%)	95% CI
288/36/36	PLS-DA	88.61 ± 5.15	[84.93%, 92.29%]
	SVM(RBF)	90.56 ± 5.11	[86.90%, 94.21%]
	FMLDA+KNN	91.39 ± 6.61	[86.66%, 96.11%]
	KNN	93.06 ± 5.75	[88.95%, 97.17%]
	Hybrid-TNet	92.50 ± 5.56	[88.52%, 96.48%]
	LightGBM	93.06 ± 6.18	[88.64%, 97.47%]
	1D-CNN	97.22 ± 2.93	[95.13%,99.32%]
	DeepSpectra	96.39 ± 3.72	[93.73%, 99.05%]
252/54/54	PLS-DA	89.81 ± 3.83	[87.07%, 92.55%]
	SVM(RBF)	90.56 ± 3.20	[88.27%, 92.85%]
	FMLDA+KNN	90.19 ± 3.71	[87.53%, 92.84%]
	KNN	92.78 ± 4.97	[89.22%, 96.34%]
	Hybrid-TNet	91.67 ± 4.72	[88.29%, 95.04%]
	LightGBM	92.41 ± 5.12	[88.74%, 96.07%]
	1D-CNN	96.11 ± 2.04	[94.65%, 97.57%]
	DeepSpectra	96.48 ± 1.84	[95.16%, 97.80%]

**Table 4:** Performance and computational complexity comparison of LightGBM, 1D-CNN, and DeepSpectra.

Performance Metrics	LightGBM	1D-CNN	DeepSpectra
Number of Parameters (10k)	−(240 trees × 31 leaves)	427.04	218.90
Floating-Point Operations (GFLOPs)	–	0.02	0.02
Inference Speed (ms/sample, single)	0.01	0.78	2.20
GPU Memory (GB)	0.00	0.074	0.072
CPU Usage (%)	31.3	11.0	11.4
RAM Usage (GB)	11.17	11.24	11.24
Deployment Hardware Requirement	General CPU device, RAM 2 GB+	Portable device, GPU 1 GB+	Portable device, GPU 1 GB+

## Data Availability

Data and the model implementation will be made available on request from the corresponding authors.
